# Blend Hydrogel Microspheres of Carboxymethyl Chitosan and Gelatin for the Controlled Release of 5-Fluorouracil

**DOI:** 10.3390/pharmaceutics9020013

**Published:** 2017-03-27

**Authors:** Vanarchi Rajini Kanth, Praveen B. Kajjari, Priya M. Madalageri, Sakey Ravindra, Lata S. Manjeshwar, Tejraj M. Aminabhavi, Vijaya Srinivasu Vallabhapurapu

**Affiliations:** 1 Department of Physics, College of Science, Engineering and Technology, University of South Africa, Pretoria 1709, South Africa; honeypree@gmail.com (P.M.M.); ravisakey@gmail.com (S.R.); Vallavs@unisa.ac.za (V.S.V.); 2 Department of Chemistry, Karnatak University, Dharwad 580 003, India; kajjari.praveen@gmail.com (P.B.K.); latamanjeshwar@yahoo.com (L.S.M.); 3 Department of Pharmaceutical Engineering and Chemistry, SET’s College of Pharmacy, Dharwad 580 002, India; aminabhavi@yahoo.com

**Keywords:** carboxymethyl chitosan, gelatin, controlled release, 5-FU, blend hydrogel microspheres

## Abstract

Carboxymethyl chitosan (CMCS) was synthesized and blended with gelatin (GE) to prepare hydrogel microspheres by w/o emulsion cross-linking in the presence of glutaraldehyde (GA), which acted as a cross-linker. 5-Fluorouracil (5-FU) was encapsulated to investigate its controlled release (CR) characteristics in acidic (pH 1.2) and alkaline (pH 7.4) buffer media. The microspheres which formed were spherical in nature, with smooth surfaces, as judged by the scanning electron microscopy (SEM). Fourier transform infrared spectroscopy (FTIR) confirmed the carboxymethylation of CS and the chemical stability of 5-FU in the formulations. Differential scanning calorimetry (DSC) and X-ray diffraction (XRD) confirmed the physical state and molecular level dispersion of 5-FU. Equilibrium swelling of microspheres was performed in water, in order to understand the water uptake properties. The in vitro release of 5-FU was extended up to 12 h in pH 7.4 phosphate buffer, revealing an encapsulation efficiency of 72%. The effects of blend composition, the extent of cross-linking, and initial drug loading on the in vitro release properties, were investigated. When analyzed through empirical equations, the release data suggested a non-Fickian transport mechanism.

## 1. Introduction

In pharmaceutical science, drug administration through an oral route is considered as the most convenient method, although it faces many hurdles relating to a harsh acidic pH and the enzymatic degradation of bioactive molecules, which in turn, reduces the effectiveness of a formulation. Attempts to maintain a constant plasma drug concentration for a longer period of time, require an increase in the dosing frequency. However, this high plasma concentration of the drug, which is higher than the required therapeutic amount, leads to severe side effects. In such situations, controlled release (CR) formulations, prepared from biodegradable polymers, offer advantages over conventional dosage forms. Biopolymeric devices not only protect the drug from systemic metabolism but also shield the tissue from direct exposure of the drug. Such biocompatible CR formulations maintain the constant therapeutic concentration of a drug for a long period of time at the site of action, thus improving the drug efficacy [1,2,3,4]. Therefore, it is important to regulate the release of a drug by developing a formulation prepared by dispersing the drug in an inert polymer matrix [5,6]. Of many such systems, formulations prepared from polymeric hydrogels are known to be effective oral CR systems as these can be developed in the form of microspheres, in which a drug can be loaded using biocompatible and biodegradable polymers to offer optimal CR characteristics for controlling the release of a drug over an extended time [7,8,9]. Biopolymers that are the natural products of living organisms or plants are relatively inexpensive and capable of a multitude of chemical modifications [10,11].

Gelatin (GE) is a well-known biodegradable and biocompatible polypeptide, derived from the biopolymer collagen. GE is edible and soluble at body temperature, but undergoes gelation at temperatures just below ambient. Due to this special nature of gelatin, it is used in pharmaceutical investigations [12,13,14,15,16]. Chitosan (CS) is derived from chitin [17]. CS is insoluble in water and most organic solvents, although it is soluble in aqueous dilute acid, and its poor solubility is a major limiting factor for developing its CR formulation [18,19,20]. If a water-soluble CS derivative can be prepared by simple chemical reactions, then the resulting derivative could be a potential candidate for developing CR formulations.

5-Fluorouracil (5-FU) has been used for the treatment of solid tumors, carcinomas of the colon or rectum, and precancerous dermatoses [21]. However, like the other drugs which are used for chemotherapy, it affects the growth of normal body cells and often causes side effects, such as hair loss, fatigue, birth defects, mouth sores, and a temporary drop in bone marrow function. To avoid such harmful side effects, it is necessary to develop CR devices for such drugs [22,23].

In the recent past, many polymeric, as well as non-polymeric, CR systems have been developed, to study their anticancer drug delivery characteristics [24,25,26]. In search of an effective biopolymeric CR device for 5-FU, to minimize its high plasma concentration-related toxic side effects, Chaturvadi et al. [27] have developed blend microspheres of poly(3-hydroxy butyrate) and cellulose acetate phthalate, and investigated their CR characteristics. Ganguly et al. [28] used the CS matrix, cross-linked with polyethylene glycol, to control the release of 5-FU by coating the CS microspheres with cellulose acetate phthalate, producing.

With the aim of continuing the search for an efficient CR polymer matrix for 5-FU, we attempt to develop 5-FU-loaded blend microspheres of carboxymethyl chitosan (CMCS) with GE, to investigate the CR properties. The drug-loaded formulations were characterized by several analytical techniques to understand their size, shape, and morphology, as well as the chemical interactions between the polymers and drug. The in vitro 5-FU release was investigated in pH 1.2 for the initial 2 h, and in pH 7.4 phosphate buffer media for up to 12 h, to study the effects of blend composition, the extent of cross-linking, and initial drug loading.

## 2. Experimental

### 2.1. Materials

Chitosan of medium molecular weight was purchased from Aldrich Chemical Co., St Louis, MO, USA; 5-FU was obtained from Sigma-Aldrich (St. Louis, MO, USA). Gelatin, monochloroacetic acid, analytical reagent grade glutaraldehyde (GA) solution 25% (*v*/*v*), *n*-hexane, and light liquid paraffin were all bought from s.d. Fine Chemicals, Maharashtra, India. Span-80 was purchased from Loba Chemicals, Maharashtra, India. Remaining reagents were used without further purification.

### 2.2. Synthesis of Carboxymethyl Chitosan (CMCS)

Chitosan (CS) was carboxymethylated as per the method reported earlier, with some minor modifications [29]. Briefly, 1g of CS was alkalized in 10 mL of a 1:1 mixture of deionized water and isopropanol, containing 4 g of NaOH. Approximately 4 g of monochloroacetic acid, dissolved in 2 mL of isopropanol, was slowly added to the mixture over a period of 30 min, at 60 °C. The reaction mixture was quenched by adding an excess of ethanol, and the solid polymer was washed repeatedly after 6 h, until the pH of the filtrate become neutral. The unreacted CS was separated by dissolving the product in water and then centrifuging the solution. Water soluble CS was precipitated in ethanol and dried.

### 2.3. Preparation of Hydrogel Microspheres

Blend hydrogel microspheres of CMCS and GE were prepared by a water-in-oil (w/o) emulsion cross-linking method, as suggested earlier [11]. A homogenous 20 mL of 14% (*w*/*v*) polymer solution was prepared in deionized water using GE and CMCS, at room temperature. A required amount of 5-FU (5, 10, or 15 wt %) was dissolved in the polymer mixture, which was slowly added to 100 mL of 2% (*w*/*v*) paraffin oil, which was stirred using a stirrer for 10 min. The calculated amount of cross linker i.e., GA (2.5, 5 or 7.5 mL), was added to the above mixture and stirred continuously, until the microspheres were obtained. These were separated by filtration and washed with *n*-hexane to remove the oil, before finally being washed with 50 mL of 0.1 M glycine solution to mask the unreacted GA. The microspheres were air-dried at 40 °C for 24 h and stored.

The assigned codes for the different formulations are listed in Table 1.

### 2.4. Drug Content and Encapsulation Efficiency

The drug concentration in each formulation was estimated by the method previously reported [12]. A total of 10 mg of the blend hydrogel microspheres were ground with an agate mortar to produce a powder, and water-soluble 5-FU was extracted in 50 mL of distilled water by sonicating the solution (UP 400s, Dr. Hielscher, GmBH, Teltow, Germany) for 60 min. To remove the polymer dust and 5-FU extract, the polymer solution was centrifuged. The clear solution was analyzed with a UV spectrophotometer (Secomam, Anthelie, Alès, France) at a λ_max_ of 266 nm.

The % drug loading and % encapsulation efficiency values were calculated as:

(1) $$\%\;5-\mathrm{FU}\;\mathrm{loading}=\frac{\mathrm{Weight}\;\mathrm{of}\;5-\mathrm{FU}\;\mathrm{in}\;\mathrm{microspheres}}{\mathrm{Weight}\;\mathrm{of}\;\mathrm{microspheres}}\times 100$$

(2) $$\%\;\mathrm{Encapsulation}\;\mathrm{efficiency}=\frac{\mathrm{Actual}\;5-\mathrm{FU}\;\mathrm{loading}}{\mathrm{Theoretical}\;5-\mathrm{FU}\;\mathrm{loading}}\times 100$$

### 2.5. Fourier Transform Infrared Spectral Measurements (FTIR)

FTIR spectra were recorded using a Nicolet (Model Impact 410, Milwaukee, WI, USA) spectrophotometer. The carboxymethylation reaction and chemical stability of 5-FU in the blend hydrogel microspheres, were analyzed using the FTIR spectra of CS, CMCS, placebo blend hydrogel microspheres, 5-FU-loaded blend hydrogel microspheres, and nascent 5-FU. Spectra were obtained by crushing the sample with KBr, forming pellets by applying 250 kg/cm^2^ pressure. The scanning was completed at a wavelength range of 4000 to 500 cm^−1^.

### 2.6. X-ray Diffraction (XRD) Study

XRD data were collected for nascent 5-FU, placebo blend hydrogel microspheres, and 5-FU-loaded blend hydrogel microspheres, to investigate the physical state of the encapsulated 5-FU in the polymer matrix. Scanning was completed up to 2θ of 80°, using a Bruker Model D8 Advance X-ray diffractometer (Bruker, Billerica, MA, USA).

### 2.7. Differential Scanning Calorimetric (DSC) Study

Differential scanning calorimetry (DSC) (Rheometric Scientific, Surrey, UK) was performed on the placebo blend hydrogel microspheres, and 5-FU-loaded microspheres, as well as on the nascent 5-FU. The sample weights, taken in the range of ~2–3 mg in aluminum cups, were heated from 10 to 400 °C at a heating rate of 10 °C/min in a nitrogen atmosphere, at a flow rate of 20 mL/min.

### 2.8. Scanning Electron Microscopic (SEM) Study

SEM micrographs of the cross-linked blend hydrogel microspheres were produced by the following procedure. To create conductive microspheres, the microspheres were coated with gold and placed on a copper stub. Scanning was completed using an SEM instrument (JEOL model 6390 LA, JEOL, Tokyo, Japan).

### 2.9. Equilibrium Swelling (ES) Study

The % equilibrium swelling of the cross-linked blend hydrogel microspheres was gravimetrically monitored in water at 37 °C. The microspheres were kept in water for 24 h to enable them to reach complete equilibrium. Excess liquid droplets were removed by tissue papers, and the swollen microspheres were weighed and dried in an oven at 40 °C for 5 h, until no change in the dry mass of the samples was observed. This permitted a calculation of the % ES, as follows:

(3) $$\%\;\mathrm{Equilibrium}\;\mathrm{swelling}=\frac{M_{s}-M_{d}}{M_{d}}\times 100$$

where *M*_s_ and *M*_d_ are the masses of the swollen and dried microspheres, respectively.

### 2.10. In Vitro 5-FU Release Study

To understand the release of 5-FU from the blend hydrogel microspheres in both simulated gastric and intestinal pH conditions, the in vitro release experiments were initially conducted in pH 1.2 for 2 h, which was then extended up to 12 h in pH 7.4 phosphate buffer media, to investigate the influence of blend composition, the extent of cross-linking, and initial drug loading. In vitro release experiments were performed in a tablet dissolution tester (LabIndia, Mumbai, India) equipped with eight baskets, at 100 rpm stirring speed. A known quantity of each sample was placed in 500 mL of dissolution media and maintained at 37 °C. At regular intervals of time, 5 mL of sample was withdrawn and analysed using a UV spectrophotometer (Secomam, Anthelie, Alès, France) at a fixed λ_max_ of 266 nm. A total of 5 mL of fresh solvent was added to the solvent media as a refill. Triplicate data were collected and cumulative release curves were drawn through the average points, with standard deviations of ±3% being included for all of the formulations.

### 2.11. Release Kinetics Analysis

To investigate the kinetics of 5-FU release from the hydrogel microspheres, in vitro release data were fitted to empirical equations, viz., Zero-order, First-order, Higuchi, and the Hixson-Crowell cube root equation, respectively, as given below [29,30].

(4) $$Q_{t}=Q_{0}-K_{0}t$$

(5) $$\ln Q_{t}=\ln Q_{0}-K_{1}t$$

(6) $$Q_{t}=K_{h}t^{1/2}$$

(7) $$Q_{t}^{1/3}=Q_{o}^{1/3}-K_{0}t$$

where *Q*_0_ is the initial amount of drug present in the hydrogel microspheres and *Q*_t_ is the amount of drug in the hydrogel microspheres after *t* hours of the dissolution experiment. The *K*_0_, *K*_1_, and *K*_h_ values are the respective rate constants. The in vitro release results have also been fitted to the equation of Korsmeyer et al. [31]:

(8) $$\frac{M_{t}}{M_{\infty }}=K_{p}t^{n}$$

where *M*_t_*/M*_∞_ is the fraction of drug released at time *t*, *K*_p_ is the rate constant, and *n* is the release exponent. The estimated values of *n* are used to assess the release mechanism.

## 3. Results and Discussions

### 3.1. Encapsulation Efficiency (EE)

The concentration of 5-FU per known mass of the microsphere is reported in terms of % EE in Table 1, for all of the formulations. These data suggest a dependence on blend composition, the amount of cross-linking agent (GA), and the extent of drug loading. The plain GE microspheres showed 76% EE, whereas plain CMCS microspheres encapsulated only 48% of the 5-FU. In the case of blend hydrogel microspheres, the % EE values ranged from 62% to 71%, i.e., blend microspheres prepared with 25 (B1), 50 (B2), and 75% (B3) (*w*/*w*) of CMCS, showed 71%, 67%, and 62% EE, respectively. This is due to the presence of CMCS in the matrix, since the presence of CMCS results in a loose network formation, which fails to encapsulate more of the drug molecules during the formulation step.

A significant effect on the % EE values of microspheres was seen upon the addition of the cross-linker, producing values of 57%, 67%, and 76%, when cross-linked with 2.5 (B4), 5 (B2), and 7.5 mL (B5) of GA, respectively. This is because the cross-linker increased the cross-link density of the matrix, so that the matrix became rigid, reducing the leaching of 5-FU from the matrix. Microspheres loaded with 5 (B2), 10 (B6), and 15% (*w*/*w*) (B7) of 5-FU, have shown 67%, 69%, and 74% EE, respectively, indicating higher EE values at a higher loading of 5-FU; at higher 5-FU loading, more molecules can be entrapped in the matrix, increasing the EE values.

### 3.2. FTIR Analysis

**Figure 1:**
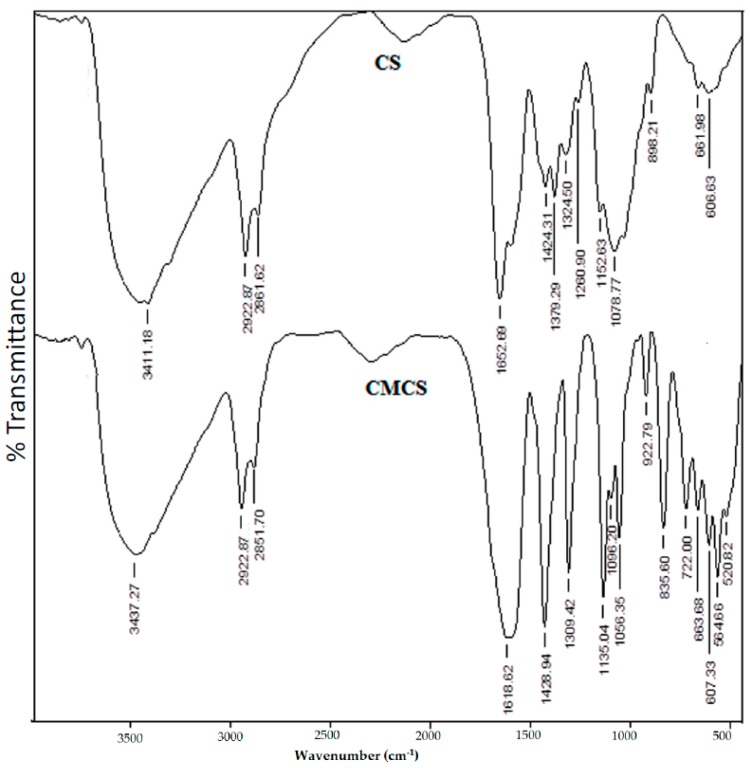
FTIR spectra of chitosan (CS) and carboxymethyl chitosan (CMCS).

The FTIR spectra of CS and CMCS are compared in Figure 1, confirming the carboxymethylation of CS. However, The FTIR spectra for CS have a C–O–C vibration frequency which appeared at 1036 cm^−1^, and the skeletal C–O–C stretching was observed at 1153 cm^−1^. The O–H stretching frequency was recorded at 3411 cm^−1^, whereas peaks observed at 1652, 1553, and 1424 cm^−1^ correspond to C=O (amide), N–H (amine), and C–N stretching frequencies, respectively.

However, the primary O–H group attached to –CH_2_OH shows the C–O stretching vibration at 1036 cm^−1^. In the case of CMCS, the O–H stretching vibration is observed at 3437 cm^−1^, whereas the peak at 1619 cm^−1^ corresponds to carboxylate C=O stretching vibrations. The amide C=O stretching frequency merges with the peak at 1619 cm^−1^ because of its broad nature. However, the amine N–H (~1553 cm^−1^) and CH_2_OH groups exhibit C–O (~1036 cm^−1^) stretching vibrations that are completely absent in the CMCS. The intensity of the band at 1429 cm^−1^, corresponding to the C–N stretching vibration, is enhanced when compared to that of CS, confirming that the amine (–NH_2_) and primary –OH groups of CS are due to the carboxymethylated sites [11].

**Figure 2:**
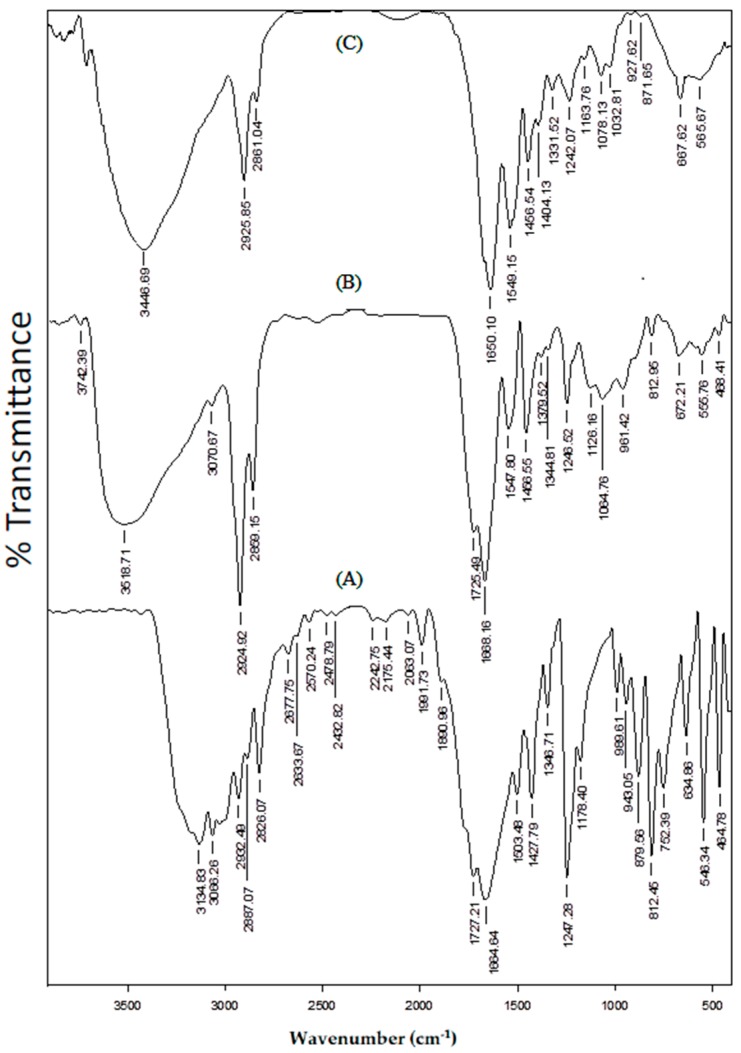
FTIR spectra of (**A**) 5-FU, (**B**) 5-FU-laded blend hydrogel microspheres, and (**C**) placeboblend hydrogel microspheres.

The FTIR spectra of (A) placebo blend hydrogel microspheres, (B) 5-FU-loaded blend hydrogel microspheres, and (C) nascent 5-FU, are compared in Figure 2, in order to understand the chemical stability of 5-FU in the formulations. In the case of 5-FU, the N–H stretching vibration appeared at 3134 cm^−1^, whereas the peaks at 3068, 2932, 2887, and 2826 cm^−1^ are attributed to both aromatic and aliphatic C–H stretching vibrations. The C=O stretching vibrations of heterocyclic imide and amide groups are observed at 1727 and 1665 cm^−1^, respectively. N–H bending is observed at 1504 cm^−1^ and C–N stretching vibrations can be seen at 1427 cm^−1^ [32]. In the case of 5-FU-loaded microspheres, along with the peaks corresponding to the placebo microspheres, the characteristic peaks of 5-FU at 3071, 1725, and 1380 cm^−1^, are also observed. The presence of unmodified functional groups of 5-FU indicates the chemical stability of 5-FU in the formulation.

### 3.3. XRD Analysis

**Figure 3:**
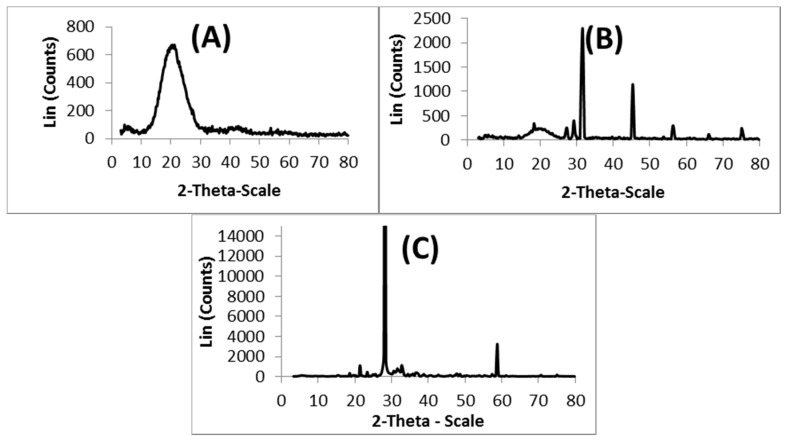
XRD patterns of (**A**) placebo blend hydrogel microspheres; (**B**) 5-FU-loaded blend hydrogel microspheres, and (**C**) 5-FU.

The XRD spectra recorded for (A) nascent 5-FU, (B) 5-FU-loaded microspheres, and (C) placebo blend hydrogel microspheres, are presented in Figure 3, to investigate the physical state of 5-FU in the matrix. The 5-FU exhibits characteristic intense peaks between 2θ of 20° and 35°, but in the case of placebo microspheres, no sharp peaks are observed between 2θ of 20° and 35°. However, with 5-FU-loaded microspheres, the characteristic peaks of 5-FU are observed along with the broad peak of the polymer matrix, indicating the crystalline nature of 5-FU in the microspheres.

### 3.4. DSC Analysis

**Figure 4:**
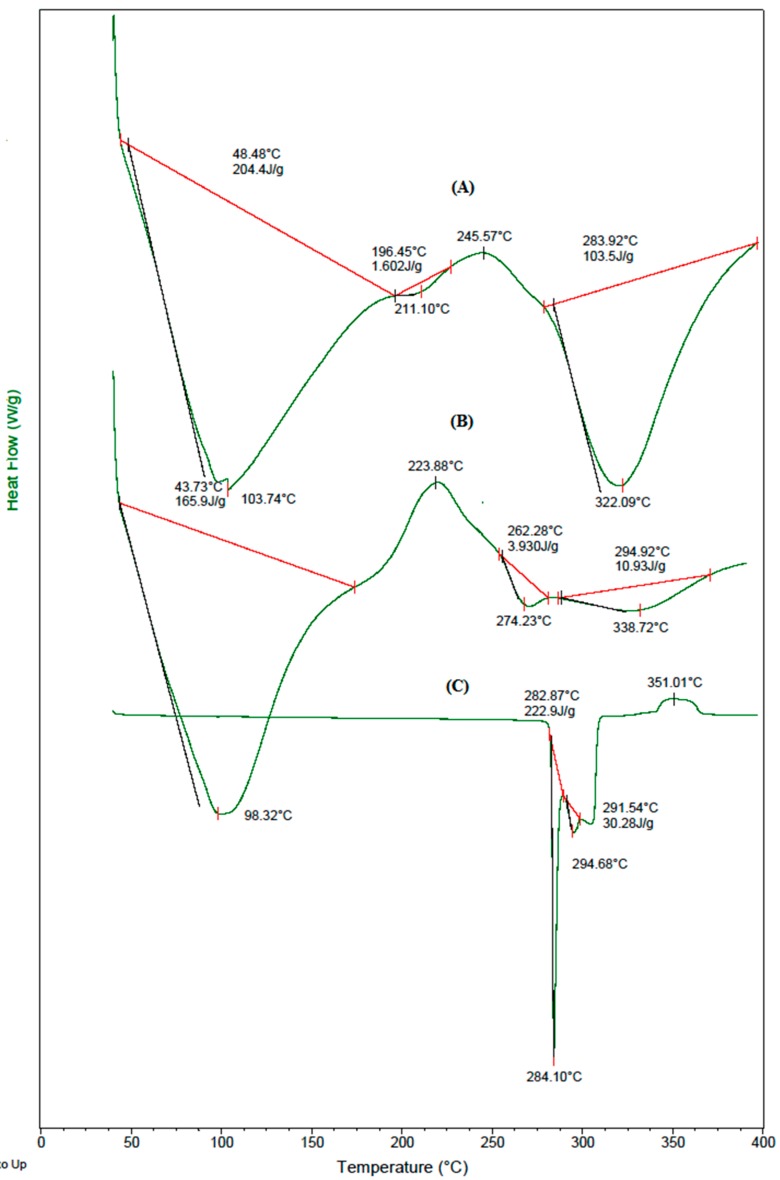
DSC thermograms of (**A**) placebo blend hydrogel microspheres; (**B**) 5-FU-loaded blend hydrogel microspheres and (**C**) 5-FU.

The DSC thermograms of (A) placebo blend hydrogel microspheres, (B) 5-FU-loaded blend hydrogel microspheres, and (C) nascent 5-FU displayed in Figure 4, show a sharp peak at 284 °C, which corresponds to its melting point. The blend microspheres underwent three endothermic transitions, and a broad endothermic peak at 104 °C is due to the loss of moisture; the peak at 322 °C suggests the degradation of GE. The phase transition occurring at 211 °C might be due to interactions between the polymeric chains. The thermogram of 5-FU-loaded blend microspheres shows a new peak at 274 °C, along with those that are present in the thermograms of placebo blend microspheres, with a slight shift; this indicates the crystalline dispersion of 5-FU in the polymer matrix, which was also previously observed in the XRD tracings.

### 3.5. SEM Analysis

**Figure 5:**
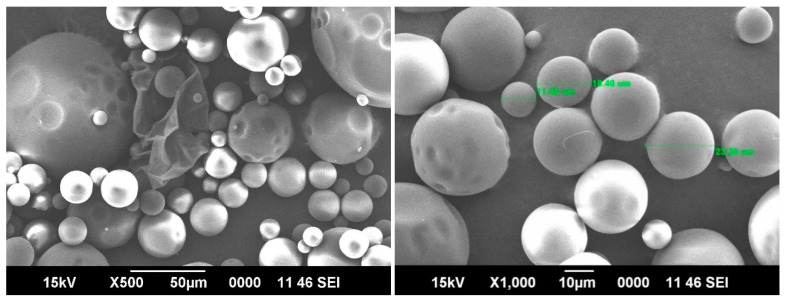
SEM micrographs of 5-FU-loaded blend hydrogel microspheres.

Typical SEM images taken for the 5-FU-loaded blend microspheres at 500× and 1000× magnifications shown in Figure 5, confirm the spherical nature of the microspheres with smooth surfaces. The hydrophilic parts of the blend microspheres shrink a little due to the loss of water during the drying process. The average range of the particles are around 20 μm. The SEM taken at 1000× magnification shows three different particle sizes, but the average size was around 20 μm.

### 3.6. Equilibrium Swelling (ES) Studies

Equilibrium water uptake of the cross-linked microspheres has an influence on the in vitro release data. The % ES data presented in Table 1 indicate 426% of ES for plain CMCS microspheres, whereas for plain GE microspheres, the ES is 328%. In the case of the blend formulation B2 containing 25% (*w*/*w*) CMCS, the % ES is 346%, whereas for the formulation B3 that contains 75% (*w*/*w*) CMCS, the % ES is 396%. On the other hand, the 50% (*w*/*w*) CMCS-containing formulation (B2), showed an intermediatory value (357%) of ES. Overall, the results of ES indicate that the formulations which contain a higher amount of CMCS, exhibit a higher level of swelling than those containing a smaller amount of CMCS; this could be due to the hydrophilic nature of CMCS. On the other hand, the formulation prepared with 2.5 mL GA cross-linker (B4) demonstrated a value of 395% ES, whereas a 308% ES was observed for the B5 formulation that was prepared with 7.5 mL GA. Such a reduction in % water uptake is due to the formation of a rigid polymer network at higher concentration of GA. As the % drug loading increased from 5% to 15% (B2, B6, and B7), the equilibrium water uptake also increased from 357% to 406%. Such a wide variation in water uptake is due to the higher dissolution of 5-FU, along with matrix swelling. When 5-FU-loaded microspheres were placed in contact with the dissolution medium, the water-soluble 5-FU diffused out of the matrix, and the diffusion media occupied the created vacant space. Therefore, high drug-loaded formulations offer more vacant spaces for the dissolution media, due to diffusion.

### 3.7. In Vitro Release Studies

To understand the in vitro release profiles of the 5-FU-loaded blend hydrogel microspheres of GE and CMCS, gastric and intestinal pH media are used to perform the release experiments. The cumulative % release vs. the time for the 5-FU-loaded microsphere formulation of B4, B2, and B5, explains the effect of crosslinking on the in vitro release study.

The formulation B4, prepared using 2.5 mL of GA, released 100% of 5-FU within 10 h of the release experiment, whereas formulation B2, prepared using 5 mL of GA, released only 85% of 5-FU. On the other hand, formulation B5, prepared with 7.5 mL of GA (B5), exhibits a value of 76% for 5-FU release. Thus, formulations prepared using a higher amount of GA show slower drug release rates than those prepared with a lower amount of GA. Also, the formulation prepared with a higher amount of GA exhibits a lower water uptake capacity, indicating that high cross-linked matrices are more rigid. Therefore, the slow release of 5-FU from the rigid microspheres is due to the slow penetration of release media into the rigid matrix, due to hindered transport into the matrix.

**Figure 6:**
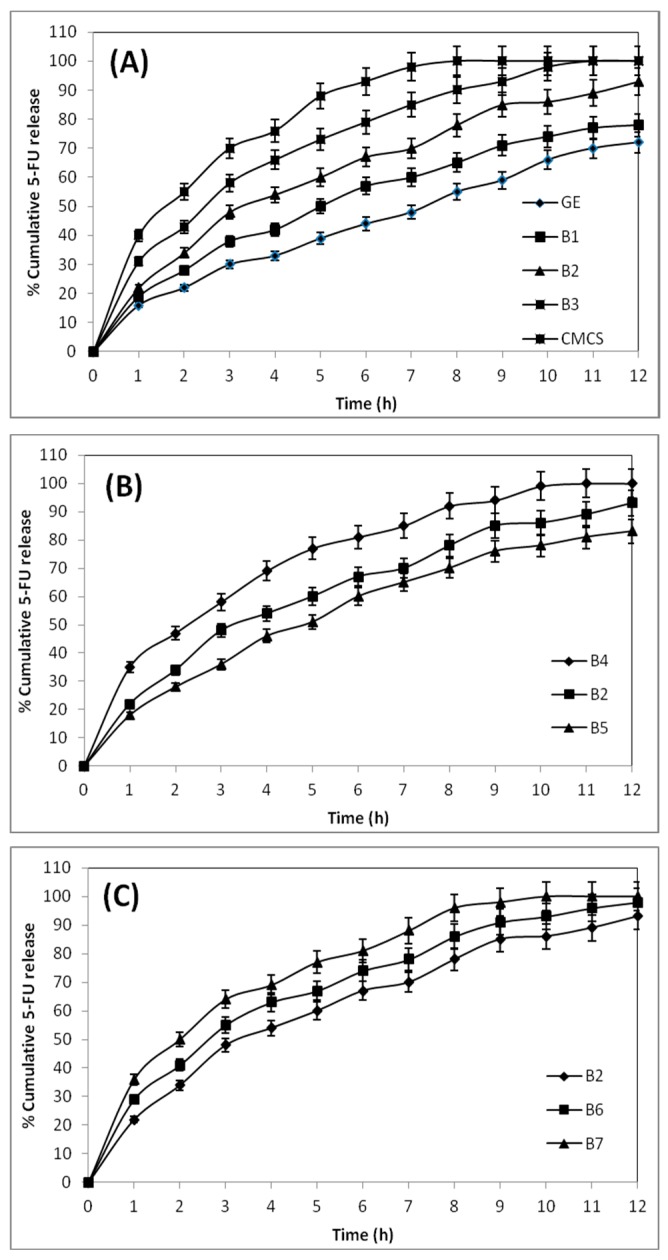
Effect of (**A**) blend composition; (**B**) amount of cross-linking agent, and (**C**) initial drug loading on in vitro 5-FU release from blend hydrogel microspheres in gastric (pH 1.2) and intestinal (pH 7.4) conditions at 37 °C.

The drug release behaviors of pristine CMCS and GE microspheres are compared with their blend hydrogel microspheres (B1, B2, and B3) in Figure 6A, to access the dependence of the drug release character of the formulations on their blend composition. The pristine CMCS microspheres released the entire encapsulated drug (100%) within 8 h, but plain GE microspheres released only 55% of 5-FU. The blend formulations viz., B1, B2, and B3, containing 25%, 50%, and 75% (*w*/*w*) of CMCS, released 66%, 78%, and 91% of 5-FU, respectively, suggesting that the blend hydrogel microspheres with a higher amount of CMCS released the encapsulated drug faster, due to the hydrophilic nature of the CMCS moiety of the blend.

The effect of drug loading on in vitro release profiles for the formulations B2, B6, and B7, displayed in Figure 6C, show a 100% release of 5-FU from B7 in 10 h, whereas the formulation B6 released 90% of 5-FU. On the other hand, B2 released 85% of 5-FU. This shows that a lower amount of drug availability results in a slower release rate for those formulations, and vice versa. When the microspheres are in contact with the release media, the surface-adhered drug molecules are first dissolved, and then the drug particle vacated sites provide a pathway for the release media to enter into the microspheres. Thus, at a higher amount of drug loading, due to the high concentration of the drug on the surface of the microspheres, a burst release occurs, producing a high % cumulative release.

### 3.8. Release Kinetics Study

The release data reported in Table 2 are fitted to zero order, First order, Higuchi square root, and Hixson-Crowell cube root equations (Equations (4)–(7)), to compute the correlation coefficients (*r*) for 5-FU release kinetics. Judging from the *r* values, the release data of all the formulations are best fitted to the Higuchi square root equation, suggesting that 5-FU release is proportional to the square root of the release time [30,31]. In order to understand the mechanism of 5-FU release, the in vitro release data were also fitted to the Korsmeyer et al. [33] Equation (Equation (8)), and the estimated *n* values, along with the correlation coefficients, are presented in Table 2. Drug release from the spherical matrix is a diffusion-controlled process if the *n* value is <0.43. The release data reported in Table 2 are fitted to zero order, First order, Higuchi square root, and Hixson-Crowell cube root equations (Equations (4)–(7)), to compute the correlation coefficients (*r*) for 5-FU release kinetics [34]. In the present formulations, the estimated *n* values for all the formulations range from 0.48 to 0.67, indicating that the release follows a non-Fickian type mechanism.

## 4. Conclusions

The present study reports on the chemical modification of the chitosan backbone to convert it into carboxymethyl chitosan, which was then blended with gelatin to prepare the hydrogel microspheres, following the w/o emulsion cross-linking method to develop oral formulations. The matrices were used to investigate the CR of 5-FU in gastrointestinal pH conditions. The spherically shaped microspheres without agglomerations are ≈20 μm in size, exhibiting varying encapsulation efficiencies of 48%–76%. However, 5-FU retained its crystalline state even after encapsulation, as evidenced by DSC and XRD. FTIR confirmed the carboxymethylation reaction of CS, forming CMCS, and the chemical stability of 5-FU in the matrix. The CMCS content of the blend affected the matrix swelling and CR of 5-FU. The estimated values of the correlation coefficients from the in vitro release data to the empirical equations indicated that the 5-FU release followed the Higuchi square root equation more than the other equations, and that the release was diffusion-controlled and non-Fickinan.

## Figures and Tables

**Table 1:** Formulation parameters of blend hydrogel microspheres of GE with CMCS along with % encapsulation efficiency (EE) and % equilibrium swelling (ES) data at 37 °C.

Formulation Codes	CMCS (% *w*/*w*)	GE (% *w*/*w*)	GA (mL)	5-FU (wt %)	EE (%)	ES (%)
GE	0	100	5	5	76	328
B1	25	75	5	5	71	346
B2	50	50	5	5	67	357
B3	75	25	5	5	62	396
CMCS	100	0	5	5	48	426
B4	50	50	2.5	5	57	395
B5	50	50	7.5	5	76	308
B6	50	50	5	10	69	388
B7	50	50	5	15	74	406

**Table 2:** Correlation coefficient (*r*) values of all the formulations estimated from different empirical equations.

Formulation Codes	Zero Order Equation (4)	First Order Equation (5)	Higuchi Equation (6)	Hixson-Crowell Equation (7)	Korsmeyer et al. Equation (8)	
					*r*	*n*
GE	0.908	0.978	0.981	0.966	0.992	0.60
B1	0.891	0.979	0.988	0.959	0.996	0.61
B2	0.904	0.980	0.985	0.963	0.991	0.64
B3	0.902	0.985	0.996	0.967	0.993	0.55
CMCS	0.903	0.976	0.997	0.943	0.993	0.48
B4	0.864	0.974	0.999	0.949	0.995	0.49
B5	0.943	0.973	0.992	0.984	0.998	0.67
B6	0.909	0.985	0.995	0.967	0.990	0.54
B7	0.839	0.961	0.996	0.929	0.993	0.48
